# Price reductions in hearing aids and access to audiologists, Israel

**DOI:** 10.2471/BLT.22.288116

**Published:** 2022-09-16

**Authors:** Limor Lavie, Tali Bar-Moshe

**Affiliations:** a Communication Sciences and Disorders, University of Haifa, 199 Aba Khoushy Ave. Mount Carmel, Haifa, 3498838, Israel.; b Department of Health Policy and Management, Ben-Gurion University of the Negev, Beer-Sheva, Israel.; Correspondence to Limor Lavie (email: llavie@welfare.haifa.ac.il).

## Abstract

**Problem:**

Hearing rehabilitation with hearing aids is a complex process which requires professional expertise and the involvement of audiologists or hearing-care specialists. Professional care, however, requires extra resources, making it tempting to rely solely on technology and reduce the role of professional counselling.

**Approach:**

To reduce the out-of-pocket share for adults needing hearing rehabilitation, in 2011 the Israeli government tripled the subsidy for adult hearing aids by converting 3 years of subsidies into a triennial, enlarged fund. Regulations for providing hearing rehabilitation and a set of rules for tenders for the supply of hearing aids were issued.

**Local setting:**

Auditory diagnosis and rehabilitation are included in the Israeli national health insurance system. Before 2011, the annual government-funded subsidy for hearing aids was negligible; hearing aids were expensive and bought mostly with patients’ own resources.

**Relevant changes:**

A series of tenders for companies to supply hearing aids, aiming to control public and individual expenses, resulted in a large reduction in prices, which in turn raised the demand for hearing aids and increased public expenditure. As the price of hearing aids fell markedly, hearing rehabilitation is approaching a point of becoming limited to supplying hearing devices, while reducing the importance placed on professional elements of the rehabilitation course.

**Lessons learnt:**

Lowering out-of-pocket costs for patients should not be the only consideration in hearing rehabilitation. Our goal should be to control public expenditure but also provide affordable hearing aids with sufficient intervention of hearing-care specialists, to ensure access to advanced technologies and proper professional care.

## Résumé

**Problème:**

La réadaptation à l'aide d'appareils auditifs est un processus complexe qui fait appel à l'expertise de professionnels et implique des audiologistes ou spécialistes de l'audition. Assurer une prise en charge adéquate nécessite toutefois des ressources supplémentaires; la tentation est donc grande de se fier uniquement à la technologie et de limiter le rôle de l'accompagnement professionnel.

**Approche:**

En vue de réduire les dépenses directes des adultes ayant besoin d'une réadaptation auditive, le gouvernement israélien a décidé, en 2011, de tripler la subvention octroyée aux appareils auditifs pour adultes, transformant trois ans de subsides en fonds triennal élargi. Des lois relatives aux services de réadaptation auditive ont été adoptées, ainsi qu'un ensemble de règles régissant les appels d'offres pour la fourniture d'appareils auditifs.

**Environnement local:**

En Israël, le diagnostic auditif et la réadaptation qui en découle font partie du régime national d'assurance-maladie. Avant 2011, le montant de la subvention annuelle financée par le gouvernement était négligeable; les appareils auditifs, coûteux, étaient généralement payés par les patients eux-mêmes.

**Changements significatifs:**

Une série d'appels d'offres ont été lancés auprès d'entreprises afin d'acquérir des prothèses auditives tout en gardant le contrôle sur les dépenses publiques et individuelles. Résultat: une forte diminution des prix qui, à son tour, a entraîné une augmentation de la demande en appareils auditifs et des dépenses publiques. Avec la chute du prix des appareils auditifs, la réadaptation se borne de plus en plus à la distribution de dispositifs d'aide à l'audition, négligeant l'importance censée être accordée à l'aspect professionnel du processus.

**Leçons tirées:**

Réduire les dépenses directes pour les patients n'est pas le seul élément à prendre en considération lorsqu'il s'agit de réadaptation auditive. Notre objectif devrait être de contrôler les dépenses publiques, mais aussi de proposer des appareils auditifs abordables assortis d'une intervention suffisante de la part des spécialistes de l'audition, afin de garantir l'accès à des technologies de pointe et des soins professionnels appropriés.

## Resumen

**Situación:**

La rehabilitación auditiva con audífonos es un proceso complejo que requiere conocimientos profesionales y la participación de los audiólogos o especialistas en atención auditiva. Sin embargo, la atención profesional requiere recursos adicionales, por lo que resulta tentador confiar únicamente en la tecnología y limitar los servicios de asesoramiento profesional.

**Enfoque:**

Para reducir la cuota de desembolso de los adultos que necesitan rehabilitación auditiva, en 2011 el gobierno israelí triplicó la subvención de los audífonos para adultos convirtiendo 3 años de subvenciones en un fondo trienal ampliado. Se publicaron reglamentos para proporcionar rehabilitación auditiva y un conjunto de normas para las licitaciones relacionadas con el suministro de audífonos.

**Marco regional:**

El diagnóstico y la rehabilitación auditiva están incluidos en el sistema nacional de seguros de salud israelí. Antes de 2011, la subvención anual que financiaba el gobierno para los audífonos era insignificante; los audífonos eran caros y se compraban en su mayoría con recursos propios de los pacientes.

**Cambios importantes:**

Gracias a una serie de licitaciones de empresas para el suministro de audífonos, con el fin de controlar los gastos públicos e individuales, se produjo una gran reducción de los precios, lo que a su vez aumentó la demanda de audífonos y el gasto público. Como el precio de los audífonos bajó de forma notable, la rehabilitación auditiva se está acercando a un punto en el que se limita a suministrar dispositivos auditivos, al tiempo que se reduce la importancia de los factores profesionales del tratamiento de rehabilitación.

**Lecciones aprendidas:**

La reducción de los gastos de bolsillo de los pacientes no debería ser el único aspecto a tener en cuenta en la rehabilitación auditiva. Nuestro objetivo debe ser controlar el gasto público, pero también proporcionar audífonos asequibles con una intervención suficiente de especialistas en audición, para garantizar el acceso a tecnologías avanzadas y a una atención profesional adecuada.

## ملخص

المشكلة إن إعادة تأهيل السمع باستخدام أدوات المساعدة السمعية هي عملية معقدة، تتطلب خبرة مهنية وإشراك أخصائيي السمع، أو متخصصي العناية بالسمع. ومع ذلك، تتطلب الرعاية المهنية مواردًا إضافية، مما يجعل من المغري الاعتماد فقط على التكنولوجيا، وتقليل دور الاستشارة المهنية.

**الأسلوب:**

لتقليص التكلفة الشخصية للبالغين الذين يحتاجون إلى إعادة تأهيل السمع، قامت الحكومة الإسرائيلية في عام 2011 بمضاعفة الدعم المخصص لأدوات المساعدة السمعية للكبار إلى ثلاثة اضعاف من خلال تحويل 3 سنوات من الدعم إلى صندوق موسع كل ثلاث سنوات. تم إصدار لوائح خاصة بتوفير إعادة تأهيل السمع، ومجموعة من القواعد الخاصة بالمناقصات المتعلقة بتوريد أدوات المساعدة السمعية.

**المواقع المحلية:**

يتم تضمين التشخيص وإعادة التأهيل السمعي في نظام التأمين الصحي الوطني الإسرائيلي. قبل عام 2011، كان الدعم السنوي الممول بواسطة الحكومة لأدوات المساعدة السمعية ضئيلًا؛ وكانت أدوات المساعدة السمعية باهظة، ويتم شراؤها في الغالب من الموارد الخاصة للمرضى.

**التغيّرات ذات الصلة:**

إن سلسلة من المناقصات للشركات لتوريد أدوات المساعدة السمعية، بهدف التحكم في النفقات العامة والفردية، أدت إلى انخفاض كبير في الأسعار، مما أدى بدوره إلى زيادة الطلب على أدوات المساعدة السمعية وزيادة الإنفاق العام. مع انخفاض سعر أدوات المساعدة السمعية بشكل ملحوظ، تقترب إعادة تأهيل السمع من أن تصبح محصورة في توفير أجهزة السمع، مع تقليل الأهمية التي توضع على العناصر المهنية لدورة إعادة التأهيل.

**الدروس المستفادة:**

لا يجب أن يكون خفض التكاليف الشخصية للمرضى هو الاعتبار الوحيد في إعادة تأهيل السمع. يجب أن يكون هدفنا هو التحكم في الإنفاق العام، وكذلك أيضًا توفير أدوات للمساعدة السمعية في متناول اليد، مع تدخل كاف من متخصصي العناية بالسمع، لضمان الوصول إلى التقنيات المتقدمة والرعاية المهنية المناسبة.

## 摘要

**问题:**

使用助听器进行听力康复治疗是一个复杂的过程，需要具备专业知识以及听力师或听力保健专家的参与。然而，专业护理由于需要额外资源的帮助，很容易完全依赖技术，使人们忽视专业咨询的作用。

**方法:**

为了减少需要进行听力康复治疗的成人患者的自付费用，以色列政府在 2011 年将成人助听器的补贴增加了两倍，将 3 年补贴转为每三年一次的扩大基金。并颁布了听力康复服务提供法规以及一系列助听器供应商招标规则。

**当地状况:**

听力诊断和康复治疗已纳入以色列国家医疗保险体系。2011 年之前，政府每年资助的助听器补贴微乎其微；助听器价格昂贵，而且大多需患者自费购买。

**相关变化:**

旨在控制公共和个人开支的一系列助听器供应商招标项目，导致助听器价格大幅下降，进而扩大了人们使用助听器的需求，并增加了公共支出。随着助听器价格显著下降，听力康复治疗正变得越来越依赖听力设备，同时，康复治疗课程的专业元素也越来越不受重视。

**经验教训:**

降低患者的自付费用不应是听力康复服务的唯一考虑因素。我们的目标应该是控制公共支出，同时提供负担得起的助听器，并由听力保健专家进行充分干预，以确保患者可以获得先进的治疗技术和适当的专业护理。

## Резюме

**Проблема:**

Восстановление слуха с использованием слуховых аппаратов представляет собой сложный процесс, требующий профессиональных знаний и участия аудиологов или специалистов по слухопротезированию. Однако для оказания профессиональной помощи требуются дополнительные ресурсы, поэтому возникает соблазн положиться исключительно на технологии и сократить долю профессионального консультирования.

**Подход:**

Чтобы уменьшить долю расходов из собственных средств для взрослых, нуждающихся в восстановлении слуха, в 2011 году правительство Израиля утроило субсидии на слуховые аппараты для взрослых путем преобразования трехлетних субсидий в расширенный на три года фонд. Изданы положения об оказании услуг по восстановлению слуха и свод правил проведения тендеров на поставку слуховых аппаратов.

**Местные условия:**

Диагностика слуха и реабилитация лиц с нарушениями слуха включены в национальную систему медицинского страхования в Израиле. До 2011 года ежегодная субсидия на слуховые аппараты, финансируемая государством, была незначительной; слуховые аппараты были дорогостоящими и покупались в основном на собственные средства пациентов.

**Осуществленные перемены:**

В результате проведения ряда тендеров среди компаний на поставку слуховых аппаратов, направленных на контроль государственных и индивидуальных расходов, произошло значительное снижение цен. В свою очередь, это привело к повышению спроса на слуховые аппараты и увеличению государственных расходов. Поскольку произошло заметное снижение цен на слуховые аппараты, процесс восстановления слуха приближается к тому моменту, когда он будет ограничиваться поставкой слуховых аппаратов. При этом снижается важность, придаваемая профессиональным элементам курса реабилитации.

**Выводы:**

Снижение расходов пациентов из собственных средств не должно стать единственным фактором при восстановлении слуха. Цель должна быть направлена не только на контроль государственных расходов, но и на обеспечение доступных слуховых аппаратов при достаточном вмешательстве специалистов по слухопротезированию, чтобы обеспечить доступ к передовым технологиям и надлежащему профессиональному уходу.

## Introduction

The World Health Organization (WHO) estimates that 466 million persons (more than 6.1% of the world’s population) live with disabling hearing loss.^1^ The prevalence of hearing loss increases with age, with the sharpest rise in adults older than 80 years.^2^ An estimated one third of adults older than 65 years are affected by hearing loss, of whom 226 million have disabling hearing loss; this number is expected to rise to 585 million by 2050.^3^ WHO estimates that more than 700 million people (one in every 10 people) will need hearing rehabilitation by 2050.^1^

Hearing rehabilitation requires both technological devices (such as hearing aids and assistive listening devices) and professional care. Rehabilitation is a complex process in which audiologists or hearing-care specialists map the communication needs and difficulties of the individual; consider multiple factors (configuration and level of hearing loss, medical comorbidities, dexterity, lifestyle and patient preferences); choose the appropriate device; discuss the patient’s expectations; solve problems; and provide listening strategies and auditory training. This professional practice aims to support continued use of the hearing devices and gain optimal benefit from their use. Hearing aids are the most common rehabilitation technological devices for people with hearing loss, but often their high price can be an obstacle to rehabilitation. Professional care also requires financial resources, which in turn raise the cost of hearing rehabilitation, making it tempting to rely solely or mainly on technology, and reduce the role of professional care.

We describe the Israeli experience, where a scheme intended to lower the out-of-pocket costs of hearing aids to patients could risk disrupting the hearing rehabilitation process.

## Local setting

The Israeli national health insurance law stipulates a set of health-care technologies and services, including auditory diagnosis and rehabilitation, which are provided to every resident of the country by four health maintenance organizations. Until 2011, the annual refund for adult hearing aids was low: up to 12% for basic technology hearing aids, and no more than 6% for high-end devices.^4^ This subsidy was negligible and not fully used; hearing aids were expensive and were bought mostly with patients’ own resources. The price of basic technology hearing aids was nearly equal to the average monthly salary, and hearing aids with more advanced technology cost around 2 months of the average wage.^5^ Indeed, in 2010 the Israeli Parliament Work, Welfare and Health Committee reported that fewer than 25 000 hearing aids were purchased in Israel every year.^6^

## Approach

In 2011, to reduce the out-of-pocket share for adult hearing rehabilitation, the Public Committee for Health Service Basket tripled the public subsidy for hearing aids for adults aged 65 years and older by converting 3 years’ subsidies into a triennial, enlarged fund of New Israeli Shekel, NIS 3000 (approximately 900 United States dollars, US$) per ear.^4^ The annual budget allocated to adult hearing aids was raised by NIS 42 million (about US$ 13 million), an increase that was estimated to cover around 20 000 hearing aids (10 000 people fitted bilaterally). Later, the eligibility was gradually broadened to all adults aged 18 years and older.^7^^–^^10^ In addition, the health authorities issued guidelines for the rehabilitation process and a set of regulations that determined rules for tenders for the supply of hearing aids, which aimed to control public and individual costs. As determined by these regulations and tenders, hearing rehabilitation with hearing aids should include the devices bundled together with a comprehensive process of counselling, fitting and follow-up provided by certified audiologists, as well as spare parts and 3.5 years full warranty on the hearing aids.^11^^,^^12^

## Relevant changes

The reform was successful, and thousands of adults who previously could not afford to purchase hearing aids were now able to have proper hearing rehabilitation. The increased demand for hearing aids, however, raised the financial load on the health maintenance organizations, requiring an increase in the budget originally allocated for hearing rehabilitation. In 2011, during the first year after the reform, the health maintenance organizations spent more than five times the allocated budget.^13^ Over the following years, the annual sales of hearing aids nearly doubled compared with before the reform, and in 2016 the Parliament Information Centre reported that more than 42 000 hearing aids were fitted annually.^7^

In attempts to control public expenses, more than a dozen tenders to companies for the supply of hearing aids were issued by the health maintenance organizations during 2011–2022. The result was intense competition among hearing aid suppliers, who greatly reduced the prices, which in turn further increased the demand for hearing aids, and consequently increased public expenditure. As the number of tenders increased, the cost of hearing aids was reduced in some of the tenders below the hearing-aid subsidy to eligible individuals. Eventually, tenders focused on the price of hearing aids but continued to require the full audiological rehabilitation and service bundle.

## Lessons learnt

As the price of hearing aids fell dramatically, hearing rehabilitation is approaching a point of becoming limited to supplying hearing aids as commodities with minimal professional care. Fortunately, the right to appropriate professional care is a core element of the Israeli medical system. Health maintenance organizations, suppliers and audiologists all insist that patients will receive the best available hearing aids and the best professional care. Our concern is that the pressure to reduce prices might result in attempts to waive the role of professional care in the rehabilitation process. Indeed, there have been attempts to deprive audiologists of essential components of their professional work.^14^ Hearing aids are classified as medical devices by many regulatory bodies,^15^^,^^16^ but when reducing the price is a major goal, it is tempting to rely solely or mainly on technology and to lessen the importance of professional components of the rehabilitation course.

The benefits of low-price hearing aids cannot be ignored, especially in low- and middle-income countries, in countries with no national health insurance or when audiology visits during hearing rehabilitation are paid for by the patient. Nevertheless, providing hearing aids at low prices, but with insufficient diagnosis, counselling and follow-up, can result in less than optimal rehabilitation and increased risk for non-use of the hearing aids.^17^

Research has demonstrated higher satisfaction and adoption rates when hearing aids were fitted according to audiology best practice as compared with self-selection of pre-programmed hearing aids.^18^ Satisfaction rates were higher even in a placebo group (no-amplification hearing aids) who interacted with a professional audiologist when receiving their devices, than in the self-selection group with no professional interaction. Audiology best practice in this study^18^ included careful fitting of the hearing aids and one orientation session with an audiologist. In Israel, practice also includes a trial period with multiple sessions dedicated to fine-tuning, problem-solving, instructions in use and communication skills training.^19^^–^^21^ This practice can enhance patient satisfaction, improve the odds of hearing aid adoption and ensure regular use of the devices.^22^ Moreover, for many individuals, self-adjustment and self-management of their devices with minimum professional guidance may be impossible.^23^

Untreated hearing loss in adults is correlated with increased odds of cognitive decline, loneliness, depression, falls and low quality of life.^24^ These outcomes can in turn result in an extra burden on both health and social budgets, emphasizing that it is essential to allocate a sufficient budget for hearing rehabilitation. In the *Guidelines for hearing aids and services for developing countries*, WHO acknowledged that while hearing aid costs should be kept low, the involvement of trained personnel in the course of hearing assessment and hearing aid fitting is an essential part of the rehabilitation process.^25^ Indeed, successful hearing health-care programmes in low- and middle-income countries not only provide affordable hearing devices but also train hearing health-care workers, raise awareness about the consequences of hearing loss and the rehabilitation process and use evidence-based fitting protocols.^26^

The Israeli reform increased the affordability of hearing aids for many individuals, but an unforeseen consequence was that regulations and multiple tenders increased bureaucracy, made the purchasing of hearing aids complicated for everyone involved and put the role of professional care at risk (Box 1). Israeli audiologists are mostly employees in private audiology clinics or in public hospitals, and only a few are self-employed. Most audiologists are supportive of the enlarged subsidy for hearing devices but are concerned about the potential ramifications of reducing the importance of professional care in the course of hearing aid fitting, with the potential undesirable consequences on their jobs and professional status. Thus, Israeli audiologists have been striving to increase awareness about the importance of professional adult hearing rehabilitation, such that government, and health-care and public authorities include the role of professional care in hearing aid fitting.^14^

Box 1 **Summary of main lessons learnt**

- Lowering the cost of hearing aids should not be the only consideration in hearing rehabilitation.
- Best practice in hearing rehabilitation with hearing aids should include professional care.
- Audiologists should be involved in decision-making when their scope of practice and professional role are discussed and decided.

Cost should not be the only consideration in hearing rehabilitation. Our goal should be to control public expenditure but also keep prices reasonable to ensure access to advanced technologies and professional care. At the same time we need to ensure that proper counselling, fitting and maintenance are conducted, and that the medical conditions, and social and personal needs of patients are considered.
